# Comparing survival functions with interval-censored data in the presence of an intermediate clinical event

**DOI:** 10.1186/s12874-018-0558-y

**Published:** 2018-10-01

**Authors:** Sohee Kim, Jinheum Kim, Chung Mo Nam

**Affiliations:** 1 Biostatistics and Computing, Yonsei University Graduate School, Seoul, Korea; 2 Department of Applied Statistics, University of Suwon, Suwon, Korea; 3 Department of Preventive Medicine/Department of Biostatistics, Yonsei University College of Medicine, Seoul, Korea

**Keywords:** Intermediate clinical event, Time-to-event, Length-biased, Interval-censored, Multiple imputation

## Abstract

**Background:**

In the presence of an intermediate clinical event, the analysis of time-to-event survival data by conventional approaches, such as the log-rank test, can result in biased results due to the length-biased characteristics.

**Methods:**

In the present study, we extend the studies of Finkelstein and Nam & Zelen to propose new methods for handling interval-censored data with an intermediate clinical event using multiple imputation. The proposed methods consider two types of weights in multiple imputation: 1) uniform weight and 2) the weighted weight methods.

**Results:**

Extensive simulation studies were performed to compare the proposed tests with existing methods regarding type I error and power. Our simulation results demonstrate that for all scenarios, our proposed methods exhibit a superior performance compared with the stratified log-rank and the log-rank tests. Data from a randomized clinical study to test the efficacy of sorafenib/sunitinib vs. sunitinib/sorafenib to treat metastatic renal cell carcinoma were analyzed under the proposed methods to illustrate their performance on real data.

**Conclusions:**

In the absence of intensive iterations, our proposed methods show a superior performance compared with the stratified log-rank and the log-rank test regarding type I error and power.

## Background

In clinical trials and longitudinal studies, a subject under study may experience an intermediate clinical event (IE) before the event of interest. The occurrence of the IE may induce changes in the survival distribution. An example of a length-biased problem due to the IE is the heart transplantation study [1]. It is necessary to know whether a heart transplant would be beneficial. The waiting time of subjects who eventually have a heart transplant must be long enough to receive treatment, whereas there is no requirement for not having a heart transplant.

To resolve length-biased problems due to the IE, the time-dependent Cox regression and landmark studies were conducted [1, 2]. The score tests based on counterfactual variables were derived by Lefkopoulou and Zelen [3] and Nam and Zelen [4]. Moreover, when the primary outcome is interval-censored, the situation is more complicated. Interval-censored data are data for which the exact failure times are not known but are known to have occurred between certain time points. Extensive studies are available regarding statistical approaches for analyzing interval-censored data. A non-parametric maximum likelihood estimation (NPMLE) of the survival function using the Newton-Rapshon algorithm has been proposed [5]. Alternatively, a self-consistent expectation maximization was suggested to compute the maximum likelihood estimators [6]. Dempster et al. [7] and Finkelstein [8] used the discrete-time proportional hazards model to implement the estimation of weighted log-rank tests for interval-censored data. A log-rank-type test was studied under the logistic model by applying Turnbull’s algorithm to estimate the pseudo-risk and failure sets [9]. Furthermore, Zhao and Sun [10] improved on the previous study by considering a multiple imputation (MI) technique to estimate the covariance matrix of the generalized log-rank statistics. A log-rank type test was proposed similar to a previous study but used different covariance matrix estimator [11]. Kim et al. [12] studied another log-rank type test that did not use an iterative algorithm. A uniform weights algorithm was proposed where a subject contributed uniformly to each mass point *s*_*k*_; point of the set, which consisted of all the distinct endpoints of the observed intervals.

A few methods have been suggested for left truncated and interval-censored (LTIC) data. Turnbull’s characterization was corrected to accommodate both truncation and interval-censoring time points [13]. It was extended to the regression model under the proportional assumption [14]. Pan and Chappell noted that NPMLE is inconsistent for the early times with LTIC data, while conditional NPMLE is consistent [15]. The estimation of the parameters in the Cox model with LTIC data and a rank-based test of survival function in LTIC were studied [16, 17]. However, the length-biased problem was not considered in those methods.

Most existing methods for interval-censored data use intensively iterative computation. To avoid this, an imputation method was considered in this study. We can obtain complete or (left-truncated and) right-censored data after imputation of the (left-truncated and) interval-censored data. Subsequently, standard statistical methods can be applied to the imputed data. For right-censored data, a semiparametric algorithm was proposed [18], motivated by the data augmentation algorithm [19]. Pan proposed a MI using Cox regression for interval-censored data by adapting previous method [20]. They repeated the algorithm until the coefficient *β*^*h*^ converged, where *h* denotes the number of iterations. A two-sample test with interval-censored data was studied via MI based on the approximate Bayesian bootstrap [21]. The MI for interval-censored data with auxiliary variables was studied [22]. Zhao and Sun [10] and Kim et al. [12] used MI techniques for computing the variance of test statistics. A log-rank test via MI was proposed [11]. After estimating the NPMLE using Turnbull’s algorithm, they imputed the exact time for all data points including right-censored data from the conditional probability of NPMLE. The methods of MI using Cox regression were extended to accommodate left-truncation [23, 24].

The purpose of this paper is to suggest new methods for analyzing LTIC data using MI.

This study is organized as follows. First, we introduce the notations and framework for interval-censored survival data. In the theoretical model and study hypotheses section, we explain a statistical procedure to compare two survival functions in the presence of the IE. Then, we propose our method with extensive simulation studies. The simulations are conducted to evaluate the properties of multiple imputation. An analysis of the Randomized Phase III SWITCH study was undertaken in the real example section, and we conclude the study with a short discussion.

## Methods

### Notation and framework

The survival time of a subject who experienced the IE implied that the survival time should exceed the waiting time for the IE. This reflects the length bias phenomenon; namely, a subject has to live long enough to experience the IE. We assume that the IE is binary and that only two treatment groups exist. Let *W* and *T* be positive real-valued random variables representing the waiting time until the occurrence of the IE and the time to an event of interest, respectively. We assume the independent of the event time *T* and waiting time *W*. Define a binary random variable *Z* to be *Z*=*I*{*W*≤*T*}. The random variables *T*_0_ and *T*_1_ are defined as the times to the event of interest conditional on *Z*=0 and 1, respectively, namely, *T*=(1−*Z*)*T*_0_+*ZT*_1_. The density probability functions of *W*, *T*_0_, and *T*_1_ are defined as *g*(*w*), *q*_0_(*t*), and *q*_1_(*t*), respectively; moreover, the corresponding survival distribution functions are *G*(*w*)=*Pr*(*W*>*w*),*Q*_0_(*t*)=*Pr*(*T*_0_>*t*), and *Q*_1_(*t*)=*Pr*(*T*_1_>*t*), respectively. The model with *Z*=1 implied that the waiting time was observed before the failure time *T*. Therefore, *T*_1_ was left truncated at the waiting time *W*. {*B*_*i*_,1≤*i*≤*N*} were considered as the truncation sets, specifically, *B*_*i*_=(*W*_*i*_,*∞*), where *N* is number of total subjects.

We further assume that the time to the event of interest *T* is interval-censored. Therefore, for the *i*th subject, we did not observe *T* exactly but observed *T*∈*A*_*i*_, where *A*_*i*_=(*L*_*i*_,*R*_*i*_] is the interval in which the event of interest occured. If *R*_*i*_=*∞*, we call it a right-censored observation. If *L*_*i*_=*R*_*i*_, we call it an exact observation. Let *δ*_*i*_=1, if the *i*th subject has experienced the event of interest; otherwise, it was considered 0. We consider the set of *N* independent pairs {*A*_*i*_,*B*_*i*_}. We assume *A*_*i*_⊆*B*_*i*_.

We now characterize the following union set $\tilde {C}^{k}$ with all observed points including left-truncated points, which may have a positive mass as mentioned by Frydman [13], where *k*=0,1. For the survival distribution of *T*_0_, *L*_*i*_ and *R*_*i*_ of a subject who does not experience the IE is included in the set $\tilde {C^{0}}$. When the IE occurs (*Z*=1), the waiting time *W* is a change point of distribution for survival. Thus, the information of the event exceeding *W* can no longer be observed. Therefore, the waiting time *W* for the IE is included in $\tilde {C^{0}}$ for *T*_0_ as the right-censoring time, but the event time exceeding *W* is not included in set $\tilde {C^{0}}$.

$$\begin{array}{@{}rcl@{}} \tilde{C^{0}} &= \{0\} \cup \{L_{i}; 1 \le i \le N, Z_{i} = 0\}\\& \cup \{R_{i}; 1 \le i \le N, Z_{i} = 0\} \cup  \\ &\quad\quad \{W_{i};1 \le i \le N, Z_{i} = 1\} \cup \{\infty \} \end{array} $$

For the survival distribution of *T*_1_, *L*_*i*_ and *R*_*i*_ of a subject who experienced the IE and the waiting time *W* as a left-truncated time are included in the set $\tilde {C^{1}}$. The subject who does not experience the IE is not included in set $\tilde {C^{1}}$.

$${\begin{aligned} \tilde{C^{1}} &=& \{0\} \cup \{L_{i}; 1 \le i \le N, Z_{i} = 1\} \cup \{R_{i}; 1 \le i \le N, Z_{i} = 1\} \cup \\ &&\{W_{i};1 \le i \le N, Z_{i} = 1\} \cup \{\infty \} \end{aligned}} $$

### Theoretical model and study hypotheses

Nam and Zelen [4] studied a length-biased problem with right-censored data in the presence of the IE. A subject who does not experience the IE means that the waiting time *W* for the IE has been right-censored; namely, *f*(*t*,*z*=0)=*q*_0_(*t*)*G*(*t*). A subject experiences the IE at *W*, the survival distribution is changed at *w* and the event occurs at *t*; namely, $f(t,w,z=1)=Q_{0}(w)g(w)\frac {q_{1}(t)}{Q_{1}(w)}$. The hypothesis *H*_0_:*q*_0*A*_(*t*)=*q*_0*B*_(*t*),*q*_1*A*_(*t*)=*q*_1*B*_(*t*) versus the general alternative, which is the complement of *H*_0_, could be considered, where *A*,*B* are two populations. Notably, the hypotheses were independent of the waiting time distribution.

They derived the score test using a proportional hazards model for comparing two sample survival functions. The score test could be written using the counting process notation. Define $\phantom {\dot {i}\!}Q_{kA}(t)=Q_{kB}(t)^{\beta _{k}}$ for *k*=0,1, *N*(*t*)=*I*(*T*≤*t*,*δ*=1),*Z*(*t*)=*I*(*W*≤*t*) and *R*(*t*)=*I*(*T*≥*t*), where *δ*=1 if observation is non-censored, and 0 otherwise. Let $s_{i} = x_{i} z_{i}(t_{i}){dN}_{i}(t_{i}), n_{i}=\sum _{j=1}^{N} x_{j} R_{j}(t_{i}) z_{j}(t_{i}),$ and $N_{i} =\sum _{j=1}^{N} R_{j}(t_{i}) z_{j}(t_{i})$, where *x*=1 if the observations were from *A*; otherwise, it was 0. The statistics $\hat {S_{1}}$ can be written as

$$\begin{array}{@{}rcl@{}} \hat{S_{1}} = \sum\limits_{i=1}^{N} x_{i} z_{i}(t_{i}) {dN}_{i}(t_{i}) - \sum\limits_{i=1}^{N} p_{i} {dN}_{i}(t_{i}), \quad p_{i}=n_{i}/N_{i} \end{array} $$

and under the null hypothesis has mean zero and variance $V\left (\hat {S_{1}}\right) = \sum _{i=1}^{N} p_{i}(1-p_{i}){dN}_{i}(t_{i})$. The statistics $\hat {S_{0}}$ can be written as

$${\begin{aligned} \hat{S}_{0} = \sum\limits_{i=1}^{N} x_{i} (1-z_{i}(t_{i})){dN}_{i}(t_{i})-\sum\limits_{i=1}^{N} \pi_{i} {dN}_{i}(t_{i}), \quad \pi_{i} =m_{i}/M_{i}, \end{aligned}} $$

where $r_{i} = x_{i} (1-z_{i}(t_{i})){dN}_{i}(t_{i}), m_{i}={\sum \nolimits }_{j=1}^{N} x_{j} R_{j}(t_{i}) (1-z_{j}(t_{i}))$, and $M_{i} ={\sum \nolimits }_{j=1}^{N} R_{j}(t_{i}) (1-z_{j}(t_{i}))$. The variance is $V\left (\hat {S_{0}}\right) = {\sum \nolimits }_{i=1}^{N} \pi _{i} (1-\pi _{i}){dN}_{i}(t_{i})$. Hence, an appropriate chi-square statistic with 2 degrees of freedom for testing *H*_0_ is given by $\chi _{2}^{2} = \hat {S_{1}^{2}}/V\left (\hat {S_{1}}\right) + \hat {S_{0}^{2}}/V\left (\hat {S_{0}}\right)$.

### Proposed methods

Multiple imputation converts interval-censored data to right-censored data so that standard methods can be applied. This method can simplify complicated situations. We propose two methods: 1) uniform weight method and 2) weighted weight method. The uniform method closely follows the method of Kim et al. [12] and the weighted method closely followed that of Huang et al. [11] to accommodate for left truncation. After imputation, the score statistics $\chi _{2}^{2}$ were used [4].

#### Uniform weight method

Kim et al. [12] assumed that the true failure time of a subject may be uniformly distributed over {*s*_*j*_,*L*_*i*_<*s*_*j*_≤*R*_*i*_, for *j*=1,...,*m*}. They calculated a pseudo-risk and failure set based on uniform weights. They used the MI techniques to estimate the variance matrix. In this study, we used the MI techniques for deriving the test statistics and their variance-covariance matrix including the imputation of a true failure time under the same assumption. We used a moderate imputation number (*M*=10) [20].Step 0: Set *r*=1, where *r* denotes an imputation number.Step 1. Characterize the set $\tilde {C_{i}^{k}}$ for each of *T*_*k*_ for *k*=0,1. The distinct endpoints set $C_{i}^{k}=\left \{s_{j}^{k}, L_{i}< s_{j}^{k} \leq R_{i}, \text { for }j = 1,..., m\right \}$ in which all the time points $\tilde {C^{k}}$ are ordered and labeled $0=s_{0}^{k} < s_{1}^{k} <... < s_{m}^{k} = \infty $ for *i*=1,...,*N*,*j*=1,...,*m*_*k*_,*k*=0,1. Step 2: If the *i*th observation is interval-censored, a value randomly sampled from a set $C_{i}^{k}$ is generated. Notably, after imputing the exact time, $T_{0}^{(r)}$ is the right-censored data, while $T_{1}^{(r)}$ is left-truncated and right-censored data. For making $T_{0}^{(r)}$, we censored the data at *W*_*i*_ if *Z*_*i*_=1. For making $T_{1}^{(r)}$, we only used the data with *Z*_*i*_=1.

$${\begin{aligned} T_{0i}^{(r)} = \left\{ \begin{array}{ll} L_{i} \quad &\text{if}~ \delta_{i} = 0, Z_{i} = 0\\ W_{i} \quad &\text{if} ~Z_{i} = 1\\ \text{sample from the set} \\ \phantom{aaaaa} \{s_{j}^{0}, L_{i}< s_{j}^{0} \leq R_{i}, \text{ for }j = 1,..., m\} \quad &\text{if} ~\delta_{i} = 1, Z_{i} = 0\\ \end{array} \right. \end{aligned}} $$

$${\begin{aligned} T_{1i}^{(r)} = \left\{ \begin{array}{ll} L_{i} \quad &\text{if}~ \delta_{i} = 0, Z_{i} = 1\\ \text{sample from the set} \\ \phantom{aaaaa} \{s_{j}^{1}, L_{i}< s_{j}^{1} \leq R_{i},\ \text{for}\ j = 1,..., m\} \quad &\text{if} ~\delta_{i} = 1, Z_{i} = 1\\ \end{array} \right. \end{aligned}} $$

Step 3. Based on the *r*th imputed (left-truncated) right-censored data, compute the Nam and Zelen’s statistics and their variance $S_{k}^{(r)}, V\left (\hat S_{k}\right)^{(r)}$ for *k*=0,1, respectively.Step 4. Repeat Steps 2 and 3 *M*(>0) times and obtain *M* pairs of $\left (S_{k}^{(r)}, V\left (\hat S_{k}\right)^{(r)}\right)$, where *r*=1,...,*M*,*k*=0,1.Step 5: Compute the sum of the average within-imputation covariance associated with *S*_*k*_ and the between-imputation variance of *S*_*k*_.

$$\begin{array}{@{}rcl@{}} \bar{S_{k}} &=& \frac{1}{M}\sum\limits_{r=1}^{M} S_{k}^{(r)},\\ V_{1}(\hat S_{k})_{mi} &\,=\,& \frac{1}{M}\sum\limits_{r=1}^{M} \hat V_{S_{k}}^{(r)} \,+\, \bigg(1\,+\,\frac{1}{M}\bigg)\frac{1}{M\,-\,1} \sum\limits_{r=1}^{M}\left(S_{k}^{(r)}\,-\,\bar{S_{k}}\right)^{2} \end{array} $$

In the present study, we applied two types of variances. The first is as described above: adding within- and between variances. The second is the subtraction of the two variances, which works well when the rate of follow-up loss is high [11]. The second term is formed as

$$\begin{array}{@{}rcl@{}} V_{2}\left(\hat S_{k}\right)_{mi}&= \frac{1}{M}{\sum\nolimits}_{r=1}^{M} \hat V_{S_{k}}^{(r)} - \frac{1}{M-1} {\sum\nolimits}_{r=1}^{M}\left(S_{k}^{(r)}-\bar{S_{k}}\right)^{2}. \end{array} $$

Thus, we can test *H*_0_ based on

$$\begin{array}{@{}rcl@{}} \chi_{2}^{2} =\bar{S_{0}}^{2} / V_{l}\left(\hat S_{0}\right)_{mi} + \bar{S_{1}}^{2} / V_{l}\left(\hat S_{1}\right)_{mi} \quad \text{for }l=1,2, \end{array} $$

where the distribution follows a chi-square with 2 degrees of freedom.

#### Weighted weight method based on NPMLE

We propose another weighted weight method based on NPMLE. We estimated the NPMLE from the original data set by Turnbull’s algorithm and used the NPMLE as weights for the imputation. The data were LTIC when having the IE; therefore, we characterized the set that may have a positive mass including truncated points, same as the above method. Step 1. Estimate the NPMLE from the original data set.Step 2. Using the NPMLE as weight, impute the data conditional on $\left \{L_{i} <T_{i}^{(r)} \leq R_{i}\right \}$.

$${\begin{aligned} T_{0i}^{(r)} = \left\{ \begin{array}{ll} L_{i} \quad &\text{if}~ \delta_{i} = 0, Z_{i} = 0\\ W_{i} \quad &\text{if}~ Z_{i} = 1\\ \text{sample from the distribution NPMLE}\\ \text{ using the NPMLE as weight} \quad &\text{if} ~\delta_{i} = 1, Z_{i} = 0\\ \end{array} \right. \end{aligned}} $$

$${\begin{aligned} T_{1i}^{(r)} = \left\{ \begin{array}{ll} L_{i} \quad &\text{if} \delta_{i} = 0, Z_{i} = 1\\ \text{sample from the distribution NPMLE}\\ \text{ using the NPMLE as weight} \quad &\text{if} \delta_{i} = 1, Z_{i} = 1\\ \end{array} \right. \end{aligned}} $$

Steps 3–5. Same as the part of the uniform weight method. Based on the *r*th imputed (left-truncated) right-censored data, we can calculate the average Nam and Zelen statistics and variance using the weighted weight method.

## Results

### Data generation

We generated the true failure time *T*_0_ and waiting time *W* from the survival distribution below: $\phantom {\dot {i}\!}Q_{0g}(t_{0})=e^{-\lambda _{0g} t}, G_{g}(w) = e^{-\mu _{g} w}$ for *g*=*A*,*B*.

Note that the probability of experiencing the IE is $\theta _{g}=\frac {\mu _{g}}{\mu _{g} + \lambda _{0g}}$. If *W*>*T*_0_, then *T*=*T*_0_. If *W*≤*T*_0_, a random variable *T*_1_ is generated from the truncated probability distribution function *q*_1*g*_(*t*)/*Q*_1*g*_(*w*) with *W*≤*T*_1_, where $\phantom {\dot {i}\!}Q_{1g}(t)=e^{-\lambda _{1g} t}$ for *g*=*A*,*B*. Therefore, *T*_1_ should be larger than *W*, so that we can generate *Q*_1*g*_(*t*)∼*U*(0,*Q*_1*g*_(*W*)). The value of *λ*_1*g*_ is chosen from the mean time to failure, *m*_1*g*_, *g*=*A*,*B*. In our simulations, *θ*_*A*_=0.5,*θ*_*B*_={0.3,0.4,0.5},*λ*_0*A*_=*λ*_0*B*_=1,*m*_1*A*_=1 and 2,*m*_1*B*_={1,1.25,1.5,2}. Define a censoring indicator *δ* that takes values 0 or 1 and follows a Bernoulli distribution with a censoring probability *c*_*p*_. *c*_*p*_ is set as 0 or 0.3. We could obtain the data set as {*T*_*i*_,*W*_*i*_,*δ*_*i*_,*Z*_*i*_,*x*_*i*_}, where *x*=1 if observations from *A*; otherwise, it was 0.

To generate interval-censored data, we first generated (*T*_*i*_,*δ*_*i*_) as above, where *T*_*i*_ and *δ*_*i*_ are independent. We assumed that each subject was scheduled to be examined at *p* different visits. The first scheduled visit time *E* is generated from *U*(0,*ψ*). For a subject having the IE, the first scheduled visit time *E* is equal to or greater than the waiting time *W*(*E*∼*U*(*W*,*W*+*ψ*)). The length of the time interval between two follow-up visits was assumed as a constant, *ψ* = 0.5. The survival time *T*_*i*_ is observed in one of intervals (0,*E*_*i*_],(*E*_*i*_,*E*_*i*_+*ψ*),...,(*E*_*i*_+*pψ*,*∞*). Let *E*_*k*_ denote the *k*th scheduled visit. At each of these time points, it was assumed that a subject could miss the scheduled visit. In such cases, *L*_*i*_ is defined as the largest follow-up visit *E*_*k*_ among scheduled visit points less the *T*_*i*_. Also, *R*_*i*_ is defined as the smallest follow-up visit *E*_*i*_ among scheduled visit points greater than *T*_*i*_. If *δ*_*i*_=0, the observation on *T*_*i*_ is right-censored. If *δ*_*i*_=1, the observation on *T*_*i*_ is observed on (*L*_*i*_,*R*_*i*_]. For right-censored data (*δ*_*i*_=0), we set *L*_*i*_ as it is, but *R*_*i*_ is set to infinity.

In the present study, we did not restrict the number of follow-up visits because a subject having the IE should survive during the waiting time and have more chance to follow up for longer. We assumed that every subject visits at the first visit time point, *E*. After that, there is a probability that a subject might not comply with the follow-up visits. We assume that a subject might miss any of the follow-up visits and is more likely to miss later visits (such as 0.1 for the first year, and 0.2 thereafter, using the Bernoulli distribution).

For comparison, we included the log-rank test and the stratified log-rank test (the stratum is experiencing the IE or not) along with our proposed tests. For the log-rank and stratified log-rank test, the true failure times were used rather than the interval-censored ones. We used two variance forms, which were formed by (1) adding and (2) subtracting within and between variance. The sample sizes were selected as 50, 100 and 200 for each group. The results reported are based on 1000 replications for each scenario.

### Simulation results

The results of the simulations are summarized from Tables 1, 2 and 3. Tables 1 and 2 show the estimate of the upper 5% of each of the five tests under the null hypothesis, whereas Table 3 shows the power under the alternative hypothesis for each scenario. The proposed methods show the appropriate 5% significant level under all scenarios. For the variance with adding form (1), the methods marginally overestimate the variance; thus, the effect sizes are less than 0.05 for most of scenarios. For the variance with subtracting form (2), the methods slightly underestimate the variance.

**Table 1:** Empirical 5%-level tests by varying *θ*_*B*_,*m*_1*A*_, and *m*_1*B*_ with *θ*_*A*_=0.5 when all events are observed in some intervals and when there are some missed visits with a probability of 0.1 for the first year and then of 0.2 thereafter

(*θ*_*A*_,*θ*_*B*_)	(*m*_0*A*_,*m*_0*B*_)	(*m*_1*A*_,*m*_1*B*_)	I	II	III-(1)	III-(2)	IV-(1)	IV-(2)
*n*=50								
(0.5, 0.5)	(1, 1)	(2, 2)	0.054	0.058	0.048	0.052	0.044	0.056
(0.5, 0.5)	(1, 1)	(1, 1)	0.055	0.050	0.042	0.052	0.044	0.053
(0.5, 0.4)	(1, 1)	(2, 2)	0.073	0.105	0.045	0.051	0.045	0.056
(0.5, 0.4)	(1, 1)	(1, 1)	0.060	0.124	0.042	0.058	0.042	0.060
(0.5, 0.3)	(1, 1)	(2, 2)	0.098	0.212	0.048	0.059	0.044	0.057
(0.5, 0.3)	(1, 1)	(1, 1)	0.057	0.236	0.046	0.057	0.047	0.055
*n*=100								
(0.5, 0.5)	(1, 1)	(2, 2)	0.051	0.048	0.051	0.058	0.052	0.058
(0.5, 0.5)	(1, 1)	(1, 1)	0.053	0.067	0.040	0.046	0.041	0.046
(0.5, 0.4)	(1, 1)	(2, 2)	0.069	0.148	0.044	0.049	0.046	0.049
(0.5, 0.4)	(1, 1)	(1, 1)	0.047	0.173	0.040	0.045	0.040	0.050
(0.5, 0.3)	(1, 1)	(2, 2)	0.137	0.372	0.049	0.056	0.050	0.060
(0.5, 0.3)	(1, 1)	(1, 1)	0.049	0.462	0.042	0.060	0.046	0.062
*n*=200								
(0.5, 0.5)	(1, 1)	(2, 2)	0.059	0.057	0.054	0.060	0.056	0.057
(0.5, 0.5)	(1, 1)	(1, 1)	0.055	0.042	0.042	0.049	0.043	0.056
(0.5, 0.4)	(1, 1)	(2, 2)	0.096	0.221	0.054	0.058	0.054	0.062
(0.5, 0.4)	(1, 1)	(1, 1)	0.061	0.282	0.045	0.053	0.044	0.052
(0.5, 0.3)	(1, 1)	(2, 2)	0.232	0.621	0.051	0.056	0.050	0.056
(0.5, 0.3)	(1, 1)	(1, 1)	0.053	0.747	0.045	0.051	0.043	0.052

I = log-rank, II = Stratified log-rank, III = Uniform weight method, IV = Weighted weight method. (1) added within and between variance, (2) subtracted within and between variance

**Table 2:** Empirical 5%-level tests by varying *θ*_*B*_,*m*_1*A*_, and *m*_1*B*_ with *θ*_*A*_=0.5 when censoring fraction is 0.3, and there are some missed visits with a probability of 0.1 for the first year and then of 0.2 thereafter

(*θ*_*A*_,*θ*_*B*_)	(*m*_0*A*_,*m*_0*B*_)	(*m*_1*A*_,*m*_1*B*_)	I	II	III-(1)	III-(2)	IV-(1)	IV-(2)
*n*=50								
(0.5, 0.5)	(1, 1)	(2, 2)	0.050	0.056	0.049	0.055	0.045	0.055
(0.5, 0.5)	(1, 1)	(1, 1)	0.065	0.060	0.044	0.058	0.043	0.055
(0.5, 0.4)	(1, 1)	(2, 2)	0.058	0.100	0.051	0.060	0.049	0.062
(0.5, 0.4)	(1, 1)	(1, 1)	0.052	0.090	0.042	0.053	0.048	0.053
(0.5, 0.3)	(1, 1)	(2, 2)	0.079	0.162	0.049	0.054	0.052	0.055
(0.5, 0.3)	(1, 1)	(1, 1)	0.047	0.200	0.048	0.058	0.043	0.054
*n*=100								
(0.5, 0.5)	(1, 1)	(2, 2)	0.052	0.055	0.045	0.049	0.048	0.051
(0.5, 0.5)	(1, 1)	(1, 1)	0.044	0.052	0.044	0.054	0.044	0.054
(0.5, 0.4)	(1, 1)	(2, 2)	0.075	0.105	0.052	0.056	0.053	0.057
(0.5, 0.4)	(1, 1)	(1, 1)	0.052	0.133	0.045	0.060	0.049	0.060
(0.5, 0.3)	(1, 1)	(2, 2)	0.110	0.258	0.046	0.058	0.046	0.054
(0.5, 0.3)	(1, 1)	(1, 1)	0.052	0.336	0.041	0.052	0.042	0.051
*n*=200								
(0.5, 0.5)	(1, 1)	(2, 2)	0.059	0.059	0.042	0.047	0.045	0.048
(0.5, 0.5)	(1, 1)	(1, 1)	0.050	0.054	0.052	0.059	0.050	0.056
(0.5, 0.4)	(1, 1)	(2, 2)	0.078	0.180	0.048	0.054	0.050	0.053
(0.5, 0.4)	(1, 1)	(1, 1)	0.057	0.219	0.044	0.050	0.043	0.051
(0.5, 0.3)	(1, 1)	(2, 2)	0.168	0.485	0.047	0.051	0.050	0.052
(0.5, 0.3)	(1, 1)	(1, 1)	0.060	0.582	0.040	0.049	0.043	0.050

I = log-rank, II = Stratified log-rank, III = Uniform weight method, IV = Weighted weight method (1) added within and between variance, (2) subtracted within and between variance

**Table 3:** Empirical power of tests by varying *m*_1*B*_ when censoring fraction is 0% and 30% and when there are some missed visits with a probability of 0.1 for the first year and then of 0.2 thereafter

(*θ*_*A*_,*θ*_*B*_)	(*m*_0*A*_,*m*_0*B*_)	(*m*_1*A*_,*m*_1*B*_)	I	II	III-(1)	III-(2)	IV-(1)	IV-(2)
Censoring fraction = 0%								
*n*=50								
(0.5, 0.5)	(1, 1)	(2, 1.5)	0.120	0.108	0.111	0.136	0.110	0.128
(0.5, 0.5)	(1, 1)	(2, 1.25)	0.222	0.181	0.250	0.283	0.245	0.281
(0.5, 0.5)	(1, 1)	(2, 1.0)	0.386	0.320	0.480	0.513	0.484	0.509
*n*=100								
(0.5, 0.5)	(1, 1)	(2, 1.5)	0.181	0.146	0.201	0.214	0.204	0.216
(0.5, 0.5)	(1, 1)	(2, 1.25)	0.373	0.315	0.471	0.501	0.474	0.505
(0.5, 0.5)	(1, 1)	(2, 1.0)	0.647	0.564	0.824	0.841	0.826	0.841
*n*=200								
(0.5, 0.5)	(1, 1)	(2, 1.5)	0.310	0.289	0.364	0.387	0.360	0.384
(0.5, 0.5)	(1, 1)	(2, 1.25)	0.652	0.575	0.808	0.821	0.812	0.821
(0.5, 0.5)	(1, 1)	(2, 1.0)	0.925	0.860	0.991	0.991	0.990	0.991
Censoring fraction = 30%								
*n*=50								
(0.5, 0.5)	(1, 1)	(2, 1.5)	0.101	0.099	0.110	0.120	0.110	0.119
(0.5, 0.5)	(1, 1)	(2, 1.25)	0.161	0.147	0.204	0.220	0.200	0.218
(0.5, 0.5)	(1, 1)	(2, 1.0)	0.266	0.229	0.388	0.417	0.391	0.414
*n*=100								
(0.5, 0.5)	(1, 1)	(2, 1.5)	0.113	0.114	0.145	0.160	0.143	0.155
(0.5, 0.5)	(1, 1)	(2, 1.25)	0.258	0.218	0.380	0.407	0.376	0.402
(0.5, 0.5)	(1, 1)	(2, 1.0)	0.474	0.400	0.707	0.724	0.704	0.723
*n*=200								
(0.5, 0.5)	(1, 1)	(2, 1.5)	0.248	0.202	0.297	0.312	0.301	0.310
(0.5, 0.5)	(1, 1)	(2, 1.25)	0.507	0.432	0.695	0.711	0.695	0.706
(0.5, 0.5)	(1, 1)	(2, 1.0)	0.802	0.720	0.957	0.960	0.956	0.959

I = log-rank, II = Stratified log-rank, III = Uniform weight method, IV = Weighted weight method (1) added within and between variance, (2) subtracted within and between variance

The stratified log-rank test was unsatisfactory if the proportion of experiencing the IE was different between the two groups (such as *θ*_*A*_ is not equal to *θ*_*B*_.). The log-rank test satisfied the nominal significance level if the survival functions were not changed after experiencing the IE regardless of the proportion. The change in survival distribution after experiencing the IE (such as, *m*_0*A*_ was not equal to *m*_1*A*_.) in addition to the difference in the proportion of the IE, which caused the log-rank test to be inappropriate. The comparison of uniform and weighted weights multiple imputation methods did not show significant differences.

When *θ*_*A*_=*θ*_*B*_=0.5, the simulation results confirmed that all tests gave the correct 5% significance level. Hence, the power calculations were restricted to this case. The value of the other parameters was *m*_0*A*_=*m*_0*B*_=1,*m*_1*A*_=2. Only the mean time to failure was changed for *m*_2*B*_. The increase in sample size or a decrease in the value of the censoring fraction *c*_*p*_ caused increase in the difference of mean time to failure, thus indicating that the power of the tests could be improved. In all cases, the proposed methods have superior power by taking advantage of the knowledge of the IE.

## Real data example

In this section, we illustrate the proposed method using real data from a randomized clinical trial evaluating the efficacy of tyrosine kinase inhibitors sorafenib and sunitinib in the treatment of patients with metastatic renal cell carcinoma. The primary endpoint was total progression-free survival (PFS), which was defined as the interval between the randomization (the start date of first-line therapy) to disease progression or death during second-line therapy. For subjects who did not switch to per-protocol second-line therapy, the first-line events were used. Subjects without tumor progression or death during second-line therapy were censored. The details of the study have been published [25].

We chose this study to illustrate our methods because it presented interesting aspects of IE. The proportion that was administered a second-line therapy was higher in sorafenib-sunitinib (So-Su) compared with sunitinib-sorafenib (Su-So) (57% vs 42%, *P* value <0.01). The total PFS and PFS of first-line treatment did not show a significant difference (So-Su vs. Su-So: 12.5 mo vs. 14.9 mo (*P* value = 0.5), 5.9 mo vs. 8.5 mo (*P* value = 0.9), respectively), whereas the PFS of second-line therapy showed a shorter duration in Su-So (5.4 mo vs. 2.8 mo, *P* value <0.001). Receiving the second-line therapy might be considered as experiencing the IE to compare the difference in survival functions by utilizing the knowledge of the proportion of having second-line therapy and the duration of first- and second-line therapy with different hazards assumption.

Since it is difficult to obtain raw data in this study, we extracted numerical data from the Kaplan–Meier (KM) graph on the total, first-line, and second-line PFS [25] by using WebPlotDigitizer v.3.9 (http://arohatgi.info/WebPlotDigitizer/). With the obtained proportion and numbers at risk tables, we can obtain the observed data as {*T*_*i*_,*W*_*i*_,*δ*_*i*_,*Z*_*i*_,*x*_*i*_} [26]. Similar KM graphs were obtained with the regenerated data. The interval of radiological assessment follow-up was 12 weeks. As in simulations, we assumed several scheduled visits and loss rates of radiological assessment to make interval-censored data of (*L*_*i*_,*R*_*i*_].

The proposed methods show a significant difference between the two arms (*P* value <0.01) unlike the log rank test and the stratified log rank test (*P* value >0.5). We also applied the methods based on the Cox model and obtained similar results [23, 24].

The hypothesis on (*β*_0_,*β*_1_) is separable as noted [4]. Therefore, we can test differences in the distributions for each parameter, namely, *H*_0_:*β*_1_=0 versus *H*_1_:*β*_1_≠0. One degree of freedom is used in a chi-square test $\chi ^{2}_{1} = \hat {S_{1}^{2}}/V\left (\hat {S_{1}}\right)$ of this hypothesis. In this case, we do not reject the null hypothesis of *β*_0_=0 (*P* value = 0.6) but reject the null hypothesis of *β*_1_=0 (*P* value <0.001), which is similar to a previous study [25].

## Discussion

We propose a general method of comparing two interval-censored samples in the presence of the IE. The occurrence of IE occurs may change the survival distribution. The focus of the current study is to compare two survival functions incorporating the information of the IE.

In the present study, we propose non-iterative multiple imputation methods for the analysis of left-truncated and interval-censored survival data. In the uniform weight method, the true failure time of a subject is assumed uniformly distributed over {*s*_*j*_,*L*_*i*_<*s*_*j*_≤*R*_*i*_, for *j*=1,...,*m*} [12]. We used an MI technique for the derivation of test statistics and its variance-covariance matrix including imputing a true failure time, while Kim et al. used a MI technique to estimate variance matrix. Uniform weight assumption in the characterized set is convenient to implement in practice. We also propose a weighted weight method based on NPMLE. After characterizing the set that may have a positive mass including truncated points [13], Turnbull’s algorithm was used to estimate the NPMLE. The performance of imputation procedures highly depends on the performance of the NPMLE. In the case of left-truncated and interval-censored data, NPMLE is not consistent, whereas conditional NPMLE is still consistent [15]. However, the problem is limited to the early time point. In the present study, we did not use any special correction because our purpose was not to obtain the exact NPMLE. The simulation did not show considerable differences compared with the uniform weight methods.

We applied the methods based on the Cox model to the real example, and the results were similar to the proposed methods [23, 24]. We applied two forms of variance that were formed by addition and subtraction. Both variance methods were efficient, but the first one was marginally overestimated, and the second one was slightly underestimated. This phenomenon is the same as described by Huang et al. [11] since the follow-up loss rate in each visit was not high.

We assumed that the IE was exactly as observed. Further studies are needed if the IE is considered as interval-censored.

## Conclusions

To avoid the length-biased problem, we recommend incorporating the information of the IE in the analysis. In the absence of intensive iterations, our proposed method exhibits a superior performance compared with the stratified log-rank and the log-rank test regarding the type I error and power.

## Abbreviations

IE: Intermediate clinical event
LTIC: Left-truncated interval-censored
MI: Multiple imputation
NPMLE: Non-parametric maximum likelihood estimation
